# Resumption of Spermatogenesis and Fertility Post Withdrawal of Hydroxyurea Treatment

**DOI:** 10.3390/ijms24119374

**Published:** 2023-05-27

**Authors:** Carlos Virgous, Letitia Lyons, Amos Sakwe, Tultul Nayyar, Shawn Goodwin, James Hildreth, Kevin Osteen, Kaylon Bruner-Tran, Oluwatobi Alawode, Phillip Bourne, Edward Richard Hills, Anthony E. Archibong

**Affiliations:** 1 Animal Care Facility, Meharry Medical College, 1005 D.B. Todd Blvd, Nashville, TN 37209, USA; 2 Department of Obstetrics and Gynecology, Meharry Medical College, 1005 D.B. Todd Blvd, Nashville, TN 37208, USA; 3 Department of Biochemistry, Cancer Biology, Neuroscience and Pharmacology, Meharry Medical College, 1005 D.B. Todd Blvd, Nashville, TN 37208, USA; 4 Department of Microbiology, Immunology and Physiology, Meharry Medical College, 1005 D.B. Todd Blvd, Nashville, TN 37208, USA; 5 Women’s Reproductive Health Research Center, Department of Obstetrics and Gynecology, Vanderbilt University School of Medicine, Nashville, TN 37232, USA

**Keywords:** hydroxyurea, male contraception, testis weight, sperm density, sperm motility, testosterone

## Abstract

Hydroxyurea (HU), a drug for treating cancers of the blood and the management of sickle cell anemia, induces hypogonadism in males. However, the impact of HU on testicular architecture and function, as well as its effects on the resumption of male fertility following treatment withdrawal, remain poorly understood. We used adult male mice to determine whether HU-induced hypogonadism is reversible. Fertility indices of mice treated with HU daily for ~1 sperm cycle (2 months) were compared with those of their control counterparts. All indices of fertility were significantly reduced among mice treated with HU compared to controls. Interestingly, significant improvements in fertility indices were apparent after a 4-month withdrawal from HU treatment (testis weight: month 1 post-HU withdrawal (M1): HU, 0.09 ± 0.01 vs. control, 0.33 ± 0.03; M4: HU, 0.26 ± 0.03 vs. control, 0.37 ± 0.04 g); sperm motility (M1: HU,12 vs. 59; M4: HU, 45 vs. control, 61%; sperm density (M1: HU, 1.3 ± 0.3 vs. control, 15.7 ± 0.9; M4: HU, 8.1 ± 2.5 vs. control, 16.8 ± 1.9 million). Further, circulating testosterone increased in the 4th month following HU withdrawal and was comparable to that of controls. When a mating experiment was conducted, recovering males sired viable offspring with untreated females albeit at a lower rate than control males (*p* < 0.05); therefore, qualifying HU as a potential candidate for male contraception.

## 1. Introduction

HU, a small molecule with many biological properties, has been a compound of scientific and clinical interest for several decades. [1,2]. This is due to its non-alkylating antineoplastic and antiviral activities that have been used for conditions in hematology, oncology, infectious disease and dermatology [3]. As an antineoplastic agent, the specific action of HU is the inhibition of ribonucleotide reductase (RR) in every living cell [3,4]. HU specifically suppresses the catalytically important tyrosyl free radical within the small subunit of RR and thus decreases the cellular dNTP levels and terminates DNA synthesis and repair and cell multiplication [5,6,7]. Consistent with this mechanism, HU slows replication forks and arrests the cell cycle in the S phase [8,9,10,11].

The termination of DNA synthesis and repair is engendered by HU reactive metabolic products resulting from its oxidation to a carbamoyl nitroso and subsequent hydrolysis to nitroxyl (HNO) and the corresponding carboxylic acid and nitric oxide (NO; [12]). The involvement of carbamoyl nitroso in the generation of reactive oxygen species (ROS; HNO and NO) beyond the threshold required for regulating cell signaling cascades induces oxidative stress (OS; [13]). Consequently, the termination of DNA synthesis by HU constitutes the molecular basis for its antitumor action [14]. According to Hassana Fathallah and George F. Atweh (2006), HU is a very well-tolerated orally administered drug that is very easy to use [15]. On the contrary, because of its chemotherapeutic properties, HU is not as safe on delicate organs such as gonads, germ cells and embryos [16,17].

According to Shin et al. (1999), single doses between 100 and 400 mg/kgof HU induced continuous dose-dependent increases in germ cell apoptosis and DNA fragmentation that peaked at 12 h prior to returning to control levels by 48 h [18]. Furthermore, chronic treatment of male rodents and men with HU triggered infertility [16,18,19,20,21,22,23] due to HU-induced OS [24]. The HU-induced infertility via OS manifests as repressed testosterone (T) secretion, reduced testis weight, sperm density and motility and increased population of abnormal morphologic forms of spermatozoa, [16,18,19,20,21]. Because the plasma membranes of germ, Sertoli and Leydig cells contain high concentrations of unsaturated fatty acids, these testicular cells are more vulnerable to HU-induced oxidative injury [25,26].

Interestingly, co-administration of Moringa leaf extract or Royal jelly (RJ; an anti-inflammatory/anti-oxidant/anti-microbial agent in worker bee glandular secretion; Refs. [27,28] with HU to adult male rats, prevented the testis from HU-induced oxidative damage [29]. These actions of Moringa leaf extract and RJ are innovative in ameliorating HU-induced testicular derangement due to continuous suppression of OS and maintenance of adequate dNTP pool in testicular proliferating tissues. However, these studies did not address the ability of moringa extract or RJ to restore testicular function after HU use as a chemotherapeutic agent. Cancer can afflict any individual and chemotherapy is usually administered for a finite period of time, after which, the patient is expected to resume proliferation of cells for normal bodily functions such as reproduction. Consequently, using a mouse model, we studied the time between HU withdrawal and the return of fertility as a prelude for determining if treatment with anti-oxidative agents (e.g., moringa leaf extract or RJ) immediately following HU withdrawal restores fertility more quickly. The objective of this study was to study the impact of HU on testicular architecture and function and subsequent resumption of fertility after HU withdrawal.

## 2. Results

### 2.1. HU Induced Changes in Testicular Weight and Content

**Figure 1:**
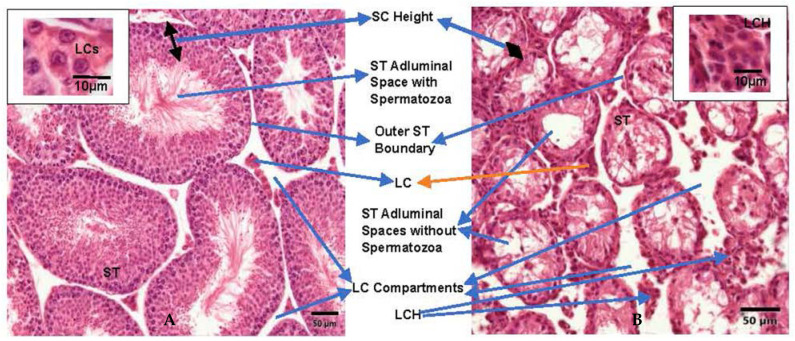
Photomicrograph of testis histology from: (**A**) control mouse; (**B**) HU-treated mouse (Magnification, 200×). HU-treated mice presented with degenerative/atrophic seminiferous tubules (ST) with only shrunken SCs and a layer of spermatogonia remaining in ST. The adluminal spaces in ST contain no spermatozoa but cellular debris. HU-treated mice also presented with enlarged Leydig cell (LC) compartments and Leydig cell hyperplasia (LCH) compared with controls. These changes indicate repressed spermatogenic activity, loss of fluid in ST and decreased testis size in HU-treated mice due to repressed T secretion.

The initial mean body weight (b wt) of mice in HU treatment group was 30.83 ± 0.91 g and comparable to that of mice in the control group (30.85 ± 0.95 g). Treatment of mice with 25 mg HU/kg did not alter the weight of the animals during the 2-month study. HU-treated versus control animals gained approximately 3.7% vs. 3.8%, respectively, of their initial b wts during the first 28 days (b wt: HU, 31.98 + 0.95; control, 32.01 + 0.59 g) and 3.6 versus 3.7% of their b wt relative to the 1st month, and at the end of the 2nd month of the study (b wt: HU, 33.13 + 0.93; control, 33.19 ± 0.89 g). Even though the dose of HU used in this study did not affect b wt, it caused an approximately 67% reduction (*p* < 0.01) in the weights of testes from the treated animals compared with those of controls at the end of the 2 months of the study (Table 1). The reduction in testis weight of treated mice was accompanied by atrophy/degeneration of seminiferous tubules (STs) which contributed to a 43% reduction in the outer ST circumference (*p* < 0.005) compared with controls (Figure 1). Our results further showed a 28% increase in the mean adluminal circumference of STs in testes recovered from HU-treated mice due in part to a 66% reduction in Sertoli cells (SCs) mean heights and the absence of spermatocytes and maturing spermatids compared with those of STs in control mice.

Interestingly, the lumen of STs of testes collected from HU-treated mice were filled with cell debris rather than mature spermatids observed in their control counterparts (Figure 1). Furthermore, HU-induced atrophic/degenerative changes in STs were accompanied by a 97% expansion (*p* < 0.005) of the LC compartment that was accompanied by a concomitant increase (*p* < 0.001) in the area occupied by LCs compared with those of controls (Figure 1). The increase in the area occupied by LCs in testes from HU-treated versus control mice suggests a HU-induced LC hyperplasia.

### 2.2. HU Induces Changes in Fertility Indices

**Figure 2:**
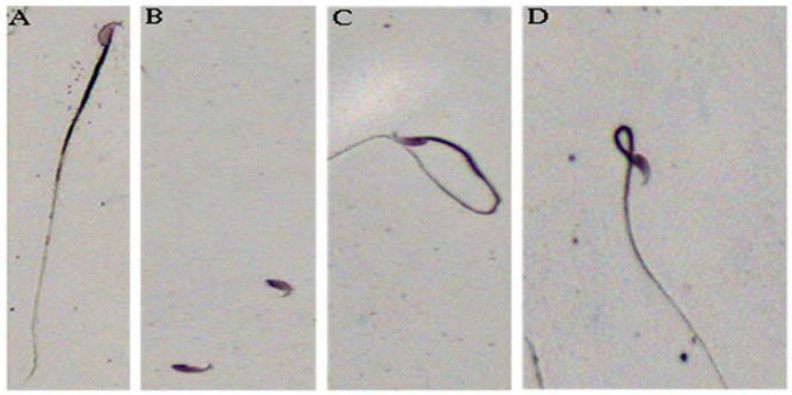
Light microscopy of normal and abnormal murine spermatozoa. All spermatozoa were classified by standard morphologic assessment. Normal spermatozoon (**A**); decapitated spermatozoa (**B**); common tail defects (**C**,**D**). Magnification, 200×.

Fertility indices of HU-treated and control mice are presented in Table 2. HU-treated mice sustained an approximately 93% reduction (*p* < 0.01) in stored sperm density compared with their control counterparts. Furthermore, the percentage of normal stored spermatozoa decreased (*p* < 0.005 (Table 2; Figure 2A), whereas that of abnormal stored spermatozoa increased (*p* < 0.01) (Table 2; Figure 2B–D) in HU-treated versus control mice.

Among spermatozoa with abnormal morphologies, decapitation (Figure 2B) was the preponderant abnormal morphologic form observed among gametes recovered from HU-treated versus their control counterparts. Other abnormal forms of spermatozoa included spermatozoa with tail abnormalities (Figure 2C,D) were also observed in spermatozoa cohorts harvested from HU-treated mice. However, their relative abundance did not differ statistically from those of spermatozoa of control mice. Furthermore, treatment of mice with HU caused a reduction (14%; *p* < 0.005) in progressive sperm motility compared with that of control mice (63%; Table 2).

Based on vaginal plug VP data, HU treatment also reduced the percentage of male mice with the ability to mate with females (percentage of females with VP: 16.7% of females caged with HU-treated males versus 100% of their control counterparts; *p* < 0.005; Table 2). Even though the number of ovulated mature ova in female mice placed with HU-treated and control males were comparable, no mature ova recovered from the only female that mated with a HU-treated male was fertilized (0%; *p* < 0.005) compared with 95% fertilization rate among controls (Table 2).

### 2.3. HU Induced Changes in Fertility Hormones

HU treatment caused an 80% reduction, a 65% and 98% increase in serum testosterone (T), luteinizing hormone (LH) and follicular stimulating hormone (FSH) concentrations, respectively, (*p* < 0.01) compared with the concentrations of similar hormones in controls. Interestingly, fertility studies conducted after HU withdrawal showed that testis weight, stored sperm motility, density and morphology remained suppressed (*p* < 0.01 (treatment x time interaction)) from the time of HU withdrawal (time 0) and 3 months later, compared with those of controls (Table 3). The suppression ranges of testis weight, sperm motility, sperm density and normal morphologic forms of spermatozoa by HU were 54–72%, 65–80%, 79–93% and 34–50%, respectively, relative to corresponding controls for the months 1, 2, 3 and 4 post-treatment withdrawal, respectively. However, by the 4th month of treatment withdrawal, sperm quality (sperm motility, density) improved (Table 3 (treatment x time interaction); *p* < 0.05) among mice previously treated with HU but remained lower (*p* < 0.05) than those of their control counterparts. Furthermore, the percentage of normal morphologic form of spermatozoa in HU-treated mice after 4 months of treatment withdrawal and that of control mice was comparable (Table 3). However, the total number of spermatozoa with normal morphology recovered from HU-treated mice after 4 months of treatment withdrawal was lower (*p* < 0.001) than that of controls (HU, 3.4 x ± 0.2; Control, 9.1 ± 0.7 million cells). Hence, mice in the HU-withdrawal group after 4 months sustained a higher level of oligo-astheno-teratozoospermia (OAT) due to reduced sperm motility, density and number of normal morphologic form of spermatozoa compared with controls.

**Figure 3:**
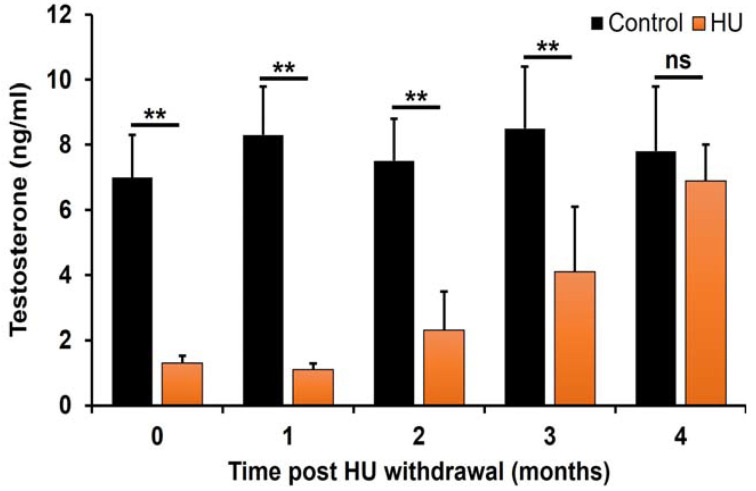
Effect of HU on serum concentrations of T in ICR male mice treated with 25 mg HU/kg. The concentrations of T were determined and compared as described in the Materials and Methods section. T concentrations among HU-treated mice were significantly lower at the end of HU treatment and 3 months thereafter (** *p* < 0.01; treatment × time interaction) compared with those of controls. However, by the 4th month, the concentrations of T were comparable (not significant; ns) in HU-treated and control mice. Data are presented as mean ± SEM (*n* = 6 pooled serum samples/treatment/control group).

T concentrations in HU-treated mice remained repressed at time 0 through the first 3 months post-HU withdrawal (*p* < 0.01; treatment × time interaction) compared to those of controls. Interestingly, mean circulating T increased in the 4th month following HU withdrawal and was comparable to that of controls (Figure 3).

**Figure 4:**
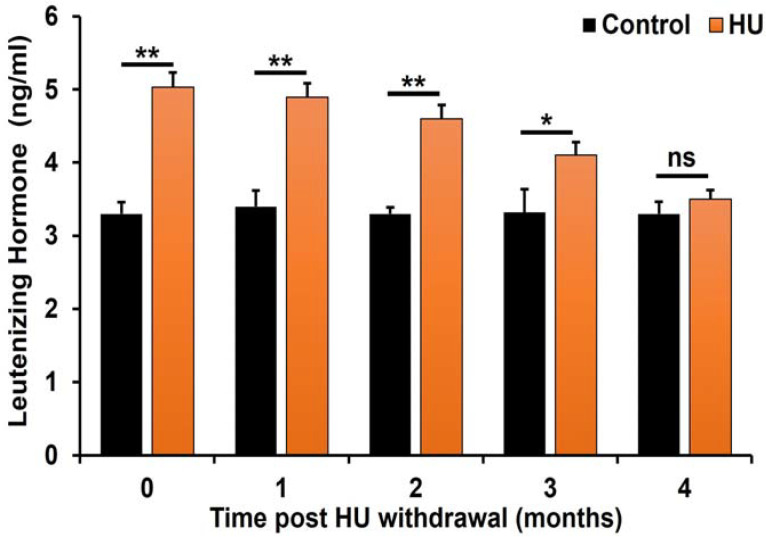
Effect of HU on serum concentrations of LH in ICR male mice treated with 25 mg HU/kg. The concentrations of LH were determined and compared as described in the Materials and Methods section. LH concentrations among HU-treated mice were significantly higher at the end of HU treatment and during the 2- and 3-month post-HU withdrawal (** *p* < 0.01 and * *p* < 0.05, respectively; treatment × time interaction) vs. controls. However, by the 4th month of post-HU withdrawal, the concentrations of LH in HU-treated and control mice were comparable (not significant; ns). Data are presented as mean ± SEM (*n* = 6 pooled serum samples/treatment/control group).

On the contrary, LH concentrations remained elevated at time 0 through the first 2 months post-HU withdrawal (*p* < 0.01 (treatment x time interaction); Figure 4) compared to those of their control counterparts. However, the levels of this gonadotropin at the 3rd month dropped by 23% (*p* < 0.05 (treatment × time interaction)) and then to comparable levels with controls in the 4th month post-HU withdrawal (Figure 4).

**Figure 5:**
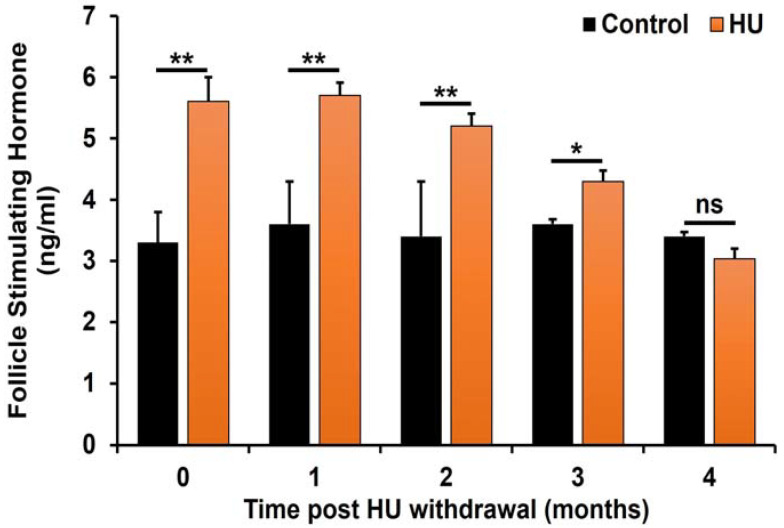
Effect of HU on serum concentrations of FSH in ICR male mice treated with 25mg HU/kg. The concentrations of FSH were determined and compared as described in the Materials and Methods section. FSH concentrations among HU-treated mice were significantly higher at the end of HU treatment and during the 2- and 3-month post-HU withdrawal (** *p* < 0.01 and * *p* < 0.05, respectively; treatment × time interaction) vs. controls. However, by the 4th month post-HU withdrawal, the concentrations of FSH in HU-treated and control mice were comparable (not significant; ns). Data are presented as mean ± SEM (*n* = 6 pooled serum samples/treatment/control group).

Similarly, FSH concentrations remained elevated at time 0 and the first 2 months of HU withdrawal (*p* < 0.01; (treatment × time interaction); Figure 5) compared with those of controls. However, on the 3rd month of HU withdrawal, FSH levels dropped to 19% (*p* < 0.05 (treatment × time interaction)) above those of controls and on the 4th month, FSH concentrations in mice previously treated with HU were comparable with those of controls.

### 2.4. Ability of Male Mice Previously Treated with HU to Sire Viable Young

**Figure 6:**
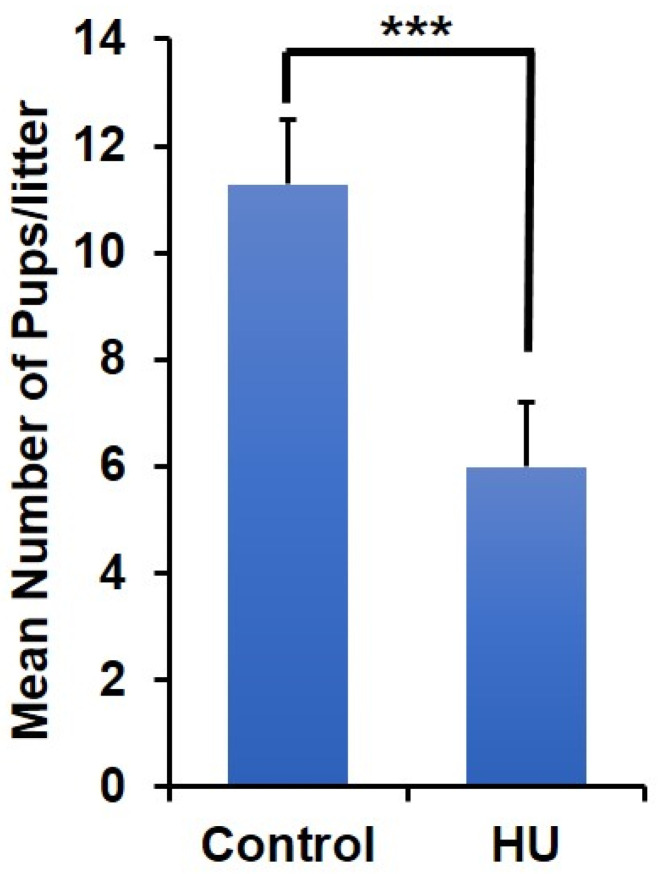
Effect of spermatozoa from ICR mice previously treated with HU on litter size of gonadotropin-stimulated female ICR mice. Bars indicate the number of pups/litter mated with male mice previously treated with vehicle control or HU. *** denotes *p* < 0.001.

Because of the improvements in fertility indices in HU-treated mice in the 4th month of HU withdrawal, it became necessary to test the fertility of these mice subsequent to HU withdrawal. After the 4th month post-HU withdrawal as described above, animals (*n* = 8/HU withdrawal or control males) were caged with gonadotropin-induced females in a 1:1 ratio. VP-positive female mice were considered as mated and observed 21 days (period of mouse gestation) later for the delivery of pups following which, litter sizes were determined 24 h post-partum. VPs were identified in six of eight and seven of eight female mice placed with males previously treated with HU and control males, respectively. The mean number (Mean ± SE) of pups per liter was reduced (*p* < 0.001) among females mated with males that had apparently recovered from HU treatment by 44% (Figure 6) compared to controls.

## 3. Discussion

HU treatment was designed to establish the adverse effects of low dose HU (25 mg/kg) used for treating sickle cell patients, on male fertility indices of wildtype male mice, as a prelude to studying fertility recovery post-treatment withdrawal. The dose of HU used in this study is equivalent to that used by us [16] in adult sickle cell male mice to induce testicular dysfunction. Furthermore, the duration of HU treatment in the present study approximates 1.5 sperm cycles (1 sperm cycle = 35 days; [16]) and was adopted to assure that spermatozoa evaluated at the end of the study originated exclusively from HU-exposed spermatogonia. Data from this study and those of others show that HU adversely affects testicular and epididymal functions regardless of the dose forms, duration of treatment and the disease being treated [3,16]. The testicular derangement observed in this study is characteristic of the typical response of the testis to chemotherapy agents, of which, HU is one. Our data showed that the regimen of HU treatment in this study adversely affected testis weight without affecting b wt. The negative relationship between testis wt and b wt in HU-treated mice in the present study has been reported by other investigators [16,30] and highlights the high sensitivity of the testis to the HU treatment. In the present study, testis wt was reduced in HU-treated mice by ~66% versus ~62% in sickle cell disease (SCD) mice [16] compared to their respective controls. Comparatively, the level of testis wt loss in HU-treated Institute of Cancer Research ICR mice and SCD mice are similar; thus, conclusions drawn on the effect of HU on the testis of one strain of mouse holds true for another mouse strain regardless of the disease modelled.

The testis bulk is comprised of proliferating and differentiating spermatogenic cells hence, HU-induced testis weight reduction should not be surprising due to the absence of secondary spermatocytes and maturing spermatids observed in this study. The absence of the above mentioned spermatogenic cells can be explained by HU-induced arrest of mitotic activity in spermatogonia at the S phase of the cell cycle [2]. Thus, arrest in mitosis of spermatogonia by HU truncates primary spermatocytes production, subsequent meiotic transformation of primary spermatogonia to secondary spermatocytes and spermatid formation [6,18]. Arguably, the expanded adluminal space in the collapsed seminiferous tubules of HU-treated mice reflects halted mitotic activity in spermatogonia and spontaneous germ cell apoptosis in premeiotic cells [18,31,32]. Further, we observed a highly significant HU-induced increase in LCC due in part to shrunken seminiferous tubules and LC hyperplasia [16,29]. LC hyperplasia is a rare benign condition that is characterized by an increased number of LCs with increased nucleoli, decreased lipofuscin and a decreased smooth endoplasmic reticulum [33,34]. LC hyperplasia usually results from an altered hypothalamic–pituitary–testicular axis, symptomatized by an elevated LH and a diminished ability of LCs to secrete T in response [33,34,35,36]. It is therefore conceivable that the repression of T secretion in HU-treated mice resulted from inhibition of two key steroidogenic enzymes (3β-HSD and P450c17) by HU-induced OS [29,37]. Consequently, the reduced circulating T in treated versus control mice, nullified the negative feedback effect of T on LH and promoted elevated LH-induced LC hyperplasia by stimulating LC progenitor proliferation [36].

The reduced Sertoli cell heights among HU-treated versus control mice in this study suggests interference in the functions of SCs. This is exemplified by elevated FSH concentration among treated versus control mice due to abrogation of negative feedback mechanism of inhibin B on FSH secretion [38]. SCs provide nutritional support and orderly structural development of germ cells to mature spermatozoa by secreting androgen binding protein (ABP) for the maintenance of high intratesticular T (ITT; [39,40,41]). High ITT concentrations are required for increased production of highly motile and normal morphologic forms of spermatozoa that can participate in fertilization events. The altered circulating T and ITT by HU-induced OS [29,37] may have contributed to reduced mating behavior and inability of the reduced mature sperm population observed in HU-treated versus control mice to participate in fertilization events.

We expected testicular steroidogenesis and spermatogenesis to resume immediately following HU withdrawal. This expectation was premised on the assumption that in vivo inhibitory effects of HU on RR are transient due to rapid HU absorption, metabolism and excretion in mammalian systems [42]. Accordingly, HU withdrawal would spontaneously lead to the regeneration of RR and the resumption of cell proliferation via reduced OS [24,43]. Unfortunately, our fertility data (hormones (FSH, LH and T secretion), sperm density and motility) did not trend to normal values within a sperm cycle post-HU withdrawal. A significant improvement in sperm density and motility of HU-treated mice were only apparent in the 4th month (~2 sperm cycles) post-HU withdrawal albeit significantly less than those of controls, whereas fertility hormone levels approximated those of controls. Interpretation of the significance of low serum T, concurrent with elevated serum LH and FSH before month 4 of HU withdrawal, is beset by the lack of knowledge of the precise threshold ITT levels required for the activation of spermatogenesis. Elevated ITT is indirectly important for the reactivation of suppressed spermatogenesis [44]. However, dihydrotestosterone (DHT), formed by the reduction of T by the 5α-reductase, has greater affinity for and a slower rate of dissociation from testicular androgen receptor and is more effective in the maintenance and reactivation of spermatogenesis than T [45,46]. It is conceivable that reduced spermatogenesis in mice in the HU-withdrawal group compared with controls may have resulted mostly from the suppression of DHT secretion via suppressed transcription of 5α-reductase [45,46] by unmitigated HU-induced OS.

The improved fertility indices after the 4th month of HU withdrawal suggests that the antioxidant status in these mice improved enough to dampen the adverse effects of HU-induced OS on testicular function. The increase in T secretion post-HU withdrawal indicates an increase in LC sensitivity to LH via the establishment of a negative feedback on LH and the reestablishment of spermatogenesis [47,48]. Further, HU withdrawal may have blunted the ability of HU-induced LC hyperplasia to repress T secretion by repopulating LCC with LCs expressing necessary key steroidogenic enzymes [29,37,45,46] to effect increased ITT and DHT secretions. Arguably, the resumption of testicular function could be hastened by reestablishing the physiologic ROS/antioxidant balance within the testis. This argument is based on the studies that show that RJ attenuates HU-induced testicular derangement [29]. The limitation to the referenced study is that the regimen of treatments did not include RJ treatment post induction of testicular injury by HU. Based on the anti-inflammatory/antioxidant properties of RJ, and in order to hasten the resumption of testicular function, it is conceivable that this product could serve as an adjuvant treatment immediately following HU treatment regimen. Interestingly, males used for mating after the period of HU withdrawal, successfully sired pups with untreated females, albeit significantly lower than the number of pups sired by control males. The reduced litter size sired by males in HU-withdrawal group may be secondary to the detrimental effects of lingering HU-induced ROS on fertility in these males [43,49]. In experiment 1, HU-treated mice lacked the ability to mate with females and produce spermatozoa that could fertilize mature eggs. The ability of males in the HU-withdrawal group to mate with females and sire pups after 4 months of treatment withdrawal indicates the resumption of fertility and predicts further improvements in fertility in the ensuing months due to improved oxidative status.

However, the reduced litter size in female mice mated with males in HU-withdrawal group compared to their control counterparts can be traced to OAT-associated subfertility among males in the HU-withdrawal group [42,50,51]. OAT is associated with increased systemic ROS which is associated with sperm damage [29] due to large amounts of oxidizable unsaturated fatty acids in their membrane, [52,53]. Therefore, some stored spermatozoa post-HU withdrawal may have ROS-damaged membrane integrity, reduced sperm motility and ultimately, the inability to acrosome react and fertilize mature ova [54,55,56].

Furthermore, reduced litter size in female mice mated to males in the HU-withdrawal group can be explained further by ROS-mediated alteration in zygote DNA and failure of embryos to develop to term. DNA of murine spermatozoa is significantly susceptible to irreparable ROS-mediated damage that perturbs paternal genetic contribution to developing embryos than nuclear DNA [57,58,59]. However, it is believed that oxidative DNA damage in spermatozoa can be repaired by maternal base excision repair pathway following fertilization (Lord and Aitken, 2015). Repair of oxidative DNA damage within the one-cell zygote, prior to the initiation of S-phase of mitosis, is a critical step in the creation of viable embryos and healthy offspring. In the absence of successful repair of oxidative lesions, such as 8-hydroxy-2’-deoxyguanosine (8OHdG) and Guanine–Cytosine (G-C) to Thymine–Adenine (T-A) transversion (Wood et al., 1992), mutations can occur during DNA replication that can alter the genetic profile of the zygote and every cell generated by the rapid mitotic divisions that characterize embryogenesis. Thus, transversion mutations within the zygote have the propensity to irreversibly alter gene expression profiles and consequently, the fidelity of normal embryonic development [60,61,62]. Thus, gametes harboring high levels of 8OHdG at fertilization, undergo inadequate DNA repair in zygotes, resulting in poor development of pre-implantation embryos [63,64] and on fetal growth and development [63,65].

Because HU treatment caused infertility in treated males which was reversed post-HU withdrawal, this qualifies HU as a potential contraceptive candidate. In experiment 1, VP was identified in one female whose mature ova were not fertilized; however, it is not apparent whether lack of fertilization is due to inability to mating due to reduced T [66]. If low T contributes to reduced mating behavior, T could be co-administered with HU to facilitate mating behavior and fulfil one of the tenets of effective contraception which is the ability to mate without effecting fertilization and pregnancy. Further studies in rodent model(s) are required before HU can be studied as a male contraceptive agent in human males. Because the dose of HU used in this study is a therapeutic dose for sickle cell disease, it will be necessary to identify a minimum effective dose of HU (MED-HU) and duration of treatment that can effectively induced infertility without preventing mating behavior. Further, for identification of the optimal time of MED-HU withdrawal that will improve fertility indices, and the ability of males to mate with females and generated normal pups/litter, a multi-generational study on pups for reproductive abnormalities will be necessary.

In conclusion, data in the present study strongly suggest that HU treatment significantly caused the derangement of testicular architecture of treated mice compared with controls. As a consequence, HU-treated mice suffered testicular failure (reduced T secretion and spermatogenesis) as well as reduced stored sperm density, motility and increased abnormal morphologic forms of spermatozoa that contributed to failed fertilization. Interestingly, there was a resurgence of spermatogenic activity in HU-treated mice at 4 months post-HU withdrawal. When these males were used to mate female mice, embryos were generated that led to the production of litters, albeit significantly reduced when compared with those generated by control males. Data presented in this study should raise awareness among adult male patients with diseases warranting the use of HU as first line treatment, and to freeze their semen prior to HU treatment, if they anticipate having a family. The results in this study suggest that HU functions to limit sperm–egg interaction by repressing the number of quality spermatozoa that could swim to the site of fertilization, and therefore, suggest that HU has the potential of serving as a male contraceptive agent. The first logical step in evaluating HU as a male contraceptive agent is to conduct mating trials with males treated with MED-HU to determine whether they can mate with females and upon HU withdrawal, when full fertility returns.

Furthermore, the ability of male mice to sire pups after HU withdrawal indicates that HU-induced infertility is tenable during treatment and shortly thereafter, thus qualifying HU as a drug that should be investigated further for possible male contraceptive development.

## 4. Materials and Methods

### 4.1. Animals

This study was approved by the Animal Care and Use Committee of Meharry Medical College, Nashville, TN. Adult male and female ICR mice, 7–8- weeks of age and weighing between 25–30 g, were purchased from Charles River Co. (Wilmington, MA, USA). Animals were housed by gender, in groups of four, in polyethylene cages and allowed to acclimatize to the animal facility environment for one week prior to initiation of studies. Animals were maintained in an environmentally controlled room with 12-h light: 12-h dark cycle (lights on at 7:00 a.m.), 22 °C and humidity range of 50 to 60% and allowed ad libitum access to commercial pelleted mouse chow and water.

### 4.2. Chemicals and Instruments

HU, pregnant mare serum gonadotropins (PMSG), human chorionic gonadotropin (hCG), M2 medium, NaHCO_3_, formalin, hyaluronidase, saline, isoflurane, 70% ethanol, 27 guage needles ), 35 × 10 Petri dishes, surgical hemostats, forceps and scissors were purchase from Sigma Aldrich (St. Louis, MO, USA). Whitten’s medium (WM) was purchased from Cosmo Bio (Carlsbad, CA, USA) and gavage needles were purchased from Kent Scientific Corporation (Torrington, CT, USA). BD vacutainer blood collection tubes were purchased from BD (Franklin Lakes, NJ, USA) Hemocytometer was purchased from American Optical (Buffalo, NY, USA). STAT III Andrology Stain was purchased from Mid-Atlantic Diagnostics, Inc (Mt Laurel, NJ, USA).

### 4.3. HU Treatment of Male Mice

Acclimated adult male mice were weighed and assigned in replicates to a treatment or a control group (*n* = 24/group). Treatment consisted of once daily dosing of 25 mg HU (dissolved in saline)/kg body weight via oral gavage for 56 days, a time period that constitutes 1.6 sperm cycles in the mice (1 sperm cycle = 35 days; [16]). Mice in the control group were similarly administered with saline (vehicle control) for the duration described for HU-treated animals and during which time, all mice were weighed every four weeks. Following the cessation of HU treatment, 36 mice (18 HU-treated and 18 control) mice were anesthetized with isoflurane to facilitate blood collection via cardiac puncture with a 27-gauge needle attached to a 1 mL tuberculin syringe. Thereafter, animals were euthanized by carbon dioxide (CO_2_) asphyxiation prior to excision of testis/epididymis complexes. The ability of HU-treated mice to mate was determined by placing the remaining control and treated mice (6/group) in a 1:1 ratio, with age- and weight-matched ICR female mice that were induced to ovulated as previously described by us [17]. Evidence of mating (presence of vaginal plug; VP in the vaginal os) was determined as described by Sampson et al., 2010 [17].

### 4.4. Post-Exposure Processing of Male Tissue Samples

Harvested blood samples were separately emptied into plus blood collection tubes (BD; Franklin Lakes, NJ) prior to serum separation by centrifugation at 3000× *g* at 4 °C for 15 min. To provide enough serum volume for hormone analyses, sera were pooled (3 samples/treatment/control group; total number of samples = 6/treatment/control group) prior to storage at −20 °C until analyzed for hormone contents. Subsequently, testis/epididymis complexes were excised and epididymides from each mouse were surgically dissected from the testes. All visible fat was trimmed from the epididymides before the caudal segments were excised for the recovery of stored spermatozoa according to the method of [67]. Briefly, both ends of each cauda epididymis were clamped with a hemostat to increase pressure within the epididymal tubules. Subsequently, each clamped cauda epididymis was placed in 1mL of M2 pre-equilibrated at 37 °C in an atmosphere of 5% CO_2_ in air under washed mineral oil in a 35 × 10 mm Petri dish (dish A: Becton Dickinson, Franklin Lakes, NJ, USA). Using a 27-gauge needle, epididymal tubules were punctured with the aid of a dissecting microscope in blood vessel-free zones to permit highly motile stored spermatozoa to escape into the medium for a duration of 5 min. Remnant cauda epididymides/mouse were transferred to a new 35 × 10 mm Petri dish (dish B; Becton Dickinson, Franklin Lakes, NJ, USA) where they were dish minced in 1 mL of pre-equilibrated M2 Medium (at conditions mentioned above) and allowed to incubate for 5 min for the remaining spermatozoa to swim out. Subsequently, cauda epididymal fragments were removed and epididymal sperm collections were assessed for sperm motility, density and morphology as described below. Testes from control and HU-treated mice were weighed prior to being fixed in 4.0% formaldehyde for 24 h. Thereafter, testes were washed twice with 70% ethanol and subsequently stored in 70% ethanol until paraffin embedded and sectioned prior to histopathology in the Meharry Translational Pathology Shared Resource Core.

### 4.5. Determination of Stored Sperm Progressive Motility and Density

Percentage sperm progressive motility was determined in a 10µL aliquot of sperm suspension from each dish A by phase contrast microscopy. Spermatozoa were considered progressively motile if they moved from one point to another. In order to determine stored sperm density per paired cauda epididymides, sperm preparations in dishes A and B were pooled per mouse and sperm density was determined by hemocytometric counting after sperm suspension were diluted 20 times with an immobilizing diluent (50 g NaHCO_3_, 10 mL of 35% (*v/v*) formalin, 5mL of saturated aqueous gentian violet and distilled water to give a final volume of 1litre [68].

### 4.6. Determination of Epididymal Sperm Morphology

A feathered smear of spermatozoa in a 20 µL aliquot from each pooled (dish A + B) sperm suspension was prepared on ethanol-cleaned microscope and allowed to dry in a dust-free area. Subsequently, spermatozoa were stained with modified Papanicolaou staining kit (STAT III Andrology Stain, Mt Laurel, NJ, USA) according to the manufacturer’s instructions and sperm morphologies were assessed according to the criteria of [69].

### 4.7. Testis Histopathology

Fixed testes from control and HU-treated rats were submitted to the Meharry Tissue Acquisition and Pathology Core laboratory for graded ethanol dehydration, paraffin wax embedding, serial sectioning (5 µm thick) and hematoxylin and eosin (H&E) staining.

### 4.8. Testicular Morphometric Analysis

Testicular morphometric analysis was conducted in the Microscopy Core laboratory of the Meharry Consolidated Research instrumentation, Informatics, Statistics, and Learning Integration Suite; CHRISALIS). Briefly, brightfield images were acquired from H&E-stained testis sections using a Nikon TE2000E microscope equipped with a Plan apo 20 × 0.75 numerical aperture lens (Nikon Instruments, Melville, NY, USA). Captured images were analyzed using Nikon Elements Advanced Research (AR) imaging software (version 4.51) with the measurements feature of the software. The 20× objective was automatically calibrated with the AR software to reveal that each pixel was 0.34 mm. Sertoli cell height was calculated using the length measurement tool and a two-point line approach. The outer circumference of seminiferous tubules and circumference of the adluminal compartment of seminiferous tubules were each calculated using the length measurement tool and the polyline feature to circumscribe the tubules. The LCCs and area occupied by LCs (a measure of hyperplasia) were calculated as described for the circumference of the seminiferous tubules. All data were exported to Excel for further analysis.

### 4.9. Fertility Evaluation of HU Exposed Male Mice

After detection of VP in female mice that were caged with HU-treated or control males, females were sacrificed by CO_2_ asphyxiation immediately at approximately 14–16 h post hCG. Subsequently, oviducts were excised via mid-ventral laparotomy and flushed with Whitten’s medium (WM) that was supplemented with 0.1% hyaluronidase and pre-equilibrated at 37 °C in an atmosphere of 5% CO_2_ in humidified air. Pooled oviductal flushing/mouse was examined with a Nikon TMD inverted microscope at 200× magnification for the presence of cumulus masses. Each pooled flushing was incubated at room temperature for approximately 10 min to permit the dispersion of cumulus cells from ovulated ova. Ova were considered mature if they each contained a nucleus devoid of germinal vesicle and had a polar body lodged in the perivitelline space. Because vaginal VP detection was conducted around the time fertilization occurs in hCG-treated mice (11 to 17 h after hCG injection; [70]), the number of fertilized ova was evaluated. An ovum was considered to be fertilized if it contained 2 pronuclei and 2 polar bodies in its cytoplasm and perivitelline space, respectively.

### 4.10. Immunoassays

Sera were analyzed in the Meharry Medical College Endocrine Core laboratory for T, FSH and LH by radioimmunoassay.

### 4.11. In Vivo HU Withdrawal Assay

HU directly inhibits RR and ultimately the depletion of cellular dNTP pools [5]; however, according to Agrawal et al. (42), spontaneous regeneration of RR occurs when HU is removed. For this reason, the in vivo effects of HU on RR are predictably transient due to rapid absorption, metabolism and excretion of HU in mammalian systems [42]. Presumably with once-daily dosing, HU should cause intermittent cytotoxic suppression of testis function, which upon withdrawal of treatment, should lead to immediate resumption of function to the testis. This experiment was designed to determine whether testicular and epididymal functions are fully restored immediately following HU withdrawal. Age and weight matched ICR male mice were procured from the vendor mentioned in experiment 1 and randomly assigned to serve as controls or receive the same dose of HU for the duration described in experiment 1. Subsequently, mice were immobilized and euthanized as described in experiment 1 at 24 h after the last HU treatment (time 0) and at 1-, 2-, 3- and 4-months post-HU withdrawal. Blood samples were collected for sera as previously described and testes/epididymal complexes were collected from mice euthanized at the five time periods post-HU withdrawal. Subsequently, testes and epididymides were separated following which, testes were weighed and cauda epididymides were used the determination of stored sperm progressive motility and density as previously described in experiment 1.

### 4.12. Statistical Analysis

In experiment 1, data on testis weight, morphometric indices, stored sperm density and serum concentrations of T, FSH, LH and number of recovered mature ova were compared by unpaired “*t*”-test. Those on stored progressive motility, normal/abnormal sperm morphology, detected VP and fertilization rate were analyzed by Chi-square. Data collected in experiment 2 (monthly body weights, testis weights, stored sperm densities and serum concentrations of T, FSH and LH post-HU withdrawal) were analyzed by Two-way analysis of variance and differences among means were tested for significance with unpaired “*t*”. Furthermore, data on monthly stored sperm progressive motility, density and normal morphology post-HU withdrawal were analyzed as described in experiment 1. Those on total number of spermatozoa with normal morphology after 4 months of HU withdrawal and litter size were compared by unpaired “t’-test. Statistical significance was defined as *p* < 0.05.

## Figures and Tables

**Table 1:** Morphometric analysis of different testicular compartments of mice treated with 25 mg/kg of hydroxyurea (HU).

Parameter	HU-Treated	Control
Testis weight (g/paired testis)	0.1 ± 0.01 **	0.30 ± 0.01 **
Outer circumference of seminiferous tubules (µm)	618 ± 39.41 ***	1082.54 ± 18.25
Adluminal circumference of seminiferous tubules (µm)	169.32 ± 11.82 **	121.48 ± 6.32
Sertoli cell height (µm)	6.1 ± 0.53 ***	17.8 ± 0.53
Area of LC compartment (µm^2^)	22175.62 ± 3390.04 ***	658.79 ± 43.26
Area occupied by LC (µm^2^)	10166.39 ± 774.42 ^¶^	1517.79 ± 175.54

^¶^ *p* < 0.001; *** *p* < 0.005; ** *p* < 0.01.

**Table 2:** Comparison of Fertility Indices (Mean + SE) of HU-treated and Control Male ICR Mice.

Item	HU Treatment	Control
Stored Sperm Density (×10^6^)	1.0 ± 0.02 ^*1^	14.1 ± 0.1
Stored Sperm Morphology (%)		
Normal	37 ^*2^	56
Abnormal	63	44
Decapitated Sperm	34 ^*2^	18
Head	14	12
Tail	15	14
Sperm Progressive Motility (%)	14.0 *^1^	63.0
Females with vaginal plug (VP; %)	16.7 *^1^	100
Mean Number of mature ova recovered	13.2 ± 2.5	12.9 ± 2.1
Fertilization rate (%)	0.0 *^1^	95
Mean T Concentrations (ng/mL)	1.5 ± 0.01 *^2^	7.4 ± 0.5
Mean LH Concentrations (ng/mL)	5.6 ± 0.4 *^2^	3.4 ± 0.1
Mean FSH Concentrations (ng/ml)	6.3 ± 1.4 *^2^	3.2 ± 0.5

*^1^ denotes *p* < 0.05; *^2^ denotes *p* < 0.01.

**Table 3:** Changes in Fertility indices in HU-treated male mice following months of HU withdrawal.

Item	Immediately After HU Withdrawal	1 Month After HU Withdrawal	2 Months After HU Withdrawal	3 Months After HU Withdrawal	4 Months After HU Withdrawal
**Testis Wt. (g)**					
Control	0.3 ± 0.01	0.33 ± 0.03	0.38 ± 0.05	0.35 ± 0.03	0.37 ± 0.04
HU	0.1 ± 0.01 **	0.09 ± 0.01 **	0.13 ± 0.04 **	0.16 ± 0.05 **	0.26 ± 0.03 *
**% Sperm Motility**					
Control	63	59	62	60	61
HU	14 **	12 **	15 **	21 **	45 *
**Stored Sperm Density (10^6^)**					
Control	14.0 ± 1.1	15.7 ± 0.9	14.9 ± 1.3	17.4 ± 1.5	16.8 ± 1.9
HU	1.0 ± 0.2 **	1.3 ± 0.3 **	1.1 ± 0.2 **	3.7 ± 2.0 **	8.1 ± 2.5 *
**Stored Sperm Morphology (%)**					
Control					
Normal	57	55	58	56	54
Abnormal	43	45	42	44	46
HU					
Normal	32	31	29	37	41
Abnormal	68	69	71	63	59

* denotes *p* < 0.05; ** denotes *p* < 0.01.

## Data Availability

The data presented in this study are available in this article.
